# Two lncRNA signatures with cuproptosis as a novel prognostic model and clinicopathological value for endometrioid endometrial adenocarcinoma

**DOI:** 10.18632/aging.205299

**Published:** 2023-12-11

**Authors:** Hongrong Wu, Ruilin Lin, Liangli Hong

**Affiliations:** 1 Department of Pathology, The First Affiliated Hospital of Shantou University Medical College, Shantou 515041, Guangdong, China

**Keywords:** cuproptosis, EEA, lncRNA

## Abstract

Objective: Cuproptosis may contribute to tumorigenesis. However, the predictive value and therapeutic significance of cuproptosis-related lncRNAs (CRLs) in endometrioid endometrial adenocarcinoma (EEA) remains unknown.

Methods: We obtained RNA-seq data from TCGA database and searched the Literature to identify cuproptosis-related genes. Using machine learning models, we identified prognostic lncRNAs for cuproptosis. Immune properties and drug sensitivity were investigated based on these signatures. Further, a ceRNA network was constructed by bioinformatics and *in vitro* experiments were performed.

Results: We determined two cuproptosis-related signatures to build the prognostic model in EEA. Afterward, the risk scores of two cuproptosis-related signatures were associated with clinicopathological molecular typing and as independent prognostic factors for EEA. In addition, we observed significant differences in immune function, checkpoints, and CD8+ T lymphocyte infiltration between the two risk groups. Furthermore, chemotherapy drugs such as AKT inhibitors exhibited lower IC50 values in the high-risk group. We speculate that ACOXL-AS1 can be served as an endogenous ‘sponge’ to regulate the expression of MTF1 by miR-421. Through *in vitro* experiments, we preliminarily validated the ceRNA network relationship in the cellular model.

Conclusion: In EEAs, this study proposed a broad molecular signature of CRLs are promising biomarkers for predicting clinical outcomes and therapeutic responses.

## INTRODUCTION

Endometrioid endometrial adenocarcinoma (EEA) is a most common histopathologic type of Uterine Corpus Endometrial Carcinoma. The prognosis of patients with endometrioid endometrial adenocarcinoma depends on several factors, such as the stage, grade, and hormone receptor status of the tumor, as well as the age and overall health of the patient [1]. Although the 5-year overall survival rate of EEA patients without metastasis is 74–91%, the prognosis of patients with EEA is not ideal [2, 3]. Therefore, it is urgent to find new treatment methods to improve the prognosis of these patients.

Copper accumulation in the human body may cause a variety of diseases. Cuproptosis, a new form of programmed cell death, may contribute to tumorigenesis [4]. Cuproptosis is a recently described type of programmed cell death, that is involved in the occurrence and development of malignant tumors [5]. Copper induces cell death by targeting fatty TCA cycle proteins [6]. Excessive intracellular copper binds to lipoacylated DLAT in cells, thereby inducing aberrant oligomerization of DLAT and formation of insoluble DLAT foci, that induce cytotoxic stress and cell death [7]. Therefore, targeting cuproptosis-related molecules may be an effective novel therapy.

Cuproptosis has been implicated in various cancers, as copper is involved in many signaling pathways and biological processes that affect tumor growth, metabolism, invasion, angiogenesis, and drug resistance. Some studies have suggested that cuproptosis may be a potential therapeutic strategy for some cancers, especially those that rely on mitochondrial respiration or have high copper levels [8].

Long non-coding RNA (lncRNA) plays a crucial role in regulating the expression of various cancer-related genes and effects on translation, histone modifications, and post-transcriptional processes [9]. It also plays important regulatory roles in posttranslational modifications, energy metabolism, cell immune and cancer patient survival [10]. Therefore, both long non-coding RNA (lncRNA) and cuproptosis are related to the regulation of energy metabolism and affect tumor progression. However, the role of cuproptosis-related lncRNAs (CRLs) in EEA remains unclear.

In this study, 2 CRLs were identified and used to construct and validate a novel prognostic signature. The correlation between signature scores and immunity, drug selection was analyzed.

## MATERIALS AND METHODS

### Publicly available datasets and preprocessing

We downloaded the mRNA expression level by RNA sequencing (RNA-Seq), somatic mutations and clinical phenotype of 516 endometrial cancer patients from TCGA database(https://portal.gdc.cancer.gov/) [11]. The RNA sequencing data was then screened against the clinical data collected for patients with endometrial cancer based on their histological subtype. Endometrioid endometrial adenocarcinoma (EEA) is a type of endometrial cancer that originates from the endometrial glands and is characterized by estrogen-dependent growth and favorable prognosis. EEA variant data were retained. RNA-Seq data were normalized using the R software package limma [12]. According to the annotation file for the platform, probes were converted into gene symbols. If several probes corresponded to the same gene symbol, the probe with the greatest value was used. The identification and removal of outliers was conducted using boxplots and principal components analyses (PCAs) [13]. Finally, the expression and clinical data of 318 patients (TCGA-UCEC) were used for the study analysis (Table 1).

**Table 1:** Baseline Characteristics of EEA patients come from TCGA.

**Characteristic**	***N* = 318^1^**
Risk	
High risk	159 (50%)
Low risk	159 (50%)
Cancer status	
Tumor free	269 (85%)
With tumor	33 (10%)
Age	
<60	133 (42%)
≥60	184 (58%)
Histologic grade	
Moderate	92 (29%)
Poor	148 (47%)
Well	78 (25%)
Myometrial invasion	
<50%	166 (58%)
≥50%	119 (42%)
Clinical stage	
Stage I–II	250 (78.5%)
Stage III–IV	68 (21.5%)
Distant metastatic	
No	307 (97%)
Yes	11 (3.5%)
Lymph modes metastatic	
No	181 (57%)
Yes	34 (11%)
Necrosis	
<10%	227 (71%)
≥10%	91 (29%)

^1^*n* (%); Median (IQR).

MAF was originally developed by The Cancer Genome Atlas (TCGA) as a standardized format for storing somatic variants detected in cancer samples, we analyze and visualize MAF Files by the MAFtools R package [14]. Tumor mutational burden (TMB) is defined as the total number of somatic, coding, base substitutions, and indels (insertions and deletions) per megabase of the genome examined, which were extracted and estimated by Perl scripts. Furthermore, the data about stemness scores, MSI status, and infiltration measure were summarized from a number of research papers, so as to provide a more comprehensive analysis. Stemness scores can be accessed at https://bioinformaticsfmrp.github.io/PanCanStem_Web [15]. The data on MSI status were acquired using the R package “TCGAbiolinks”. Infiltration measure data (ESTIMATE, CIBERSORT etc.) were obtained by an R package ‘IOBR’ [16].

### Coexpression analysis between cuproptosis-related genes and lncRNAs

By reviewing the literature, we obtained 19 key genes including NFE2L2, NLRP3, ATP7B, ATP7A, SLC31A1, FDX1, LIAS, LIPT1, LIPT2, DLD, DLAT, PDHA1, PDHB, MTF1, GLS, CDKN2A, DBT, GCSH, DLST associated with copper death signaling [17]. Then, we performed coexpression analysis between cuproptosis-related genes and lncRNAs using the “correplot” package in R [12]. Co-expressed genes can be identified by using the Pearson correlation coefficient with a threshold of at least 0.4 and a *p*-value lower than 0.001. R package “ggalluvial” was used to visualize the results. Finally, 941 cuproptosis-related lncRNAs were found.

### Identification of cuproptosis-associated clusters and survival analysis

After performing univariate Cox regression analysis of CRLs, we retained 11 lncRNAs associated with cuproptosis. ConsensusClusterPlus R package was used to analyze consistency [18]. The maximum number of clusters was set to 6, and 100 samples were drawn for clusterAlg = “hc”, and innerLinkage = “average”. Then, we performed a survival analysis for the clusters.

### Prognostic model construction and Nomogram prediction model

A risk signature for predicting the prognosis of EEA patients was constructed based on the lncRNAs related to cuproptosis. Least absolute shrinkage and selection operator (LASSO) regression was used to select predictors to avoid overfitting [19]. Multivariate Cox regression analysis was used to determine the candidate genes for final inclusion in the risk model. A risk signature for predicting the prognosis of EEA patients was constructed based on the lncRNAs related to cuproptosis. The mathematical formula for calculating the risk score was as follows:


$$\text{riskscore}=\sum_{i=1}^{N}Expression\_i\;\times \;coefficient\_i$$

Patients were divided into high and low risk groups using the median risk score as the cutoff value.

Nomograms are widely used for cancer prognosis mainly because of their ability to reduce statistical predictive models to a single numerical estimate of event probabilities [20]. Based on the results of LASSO and multivariate COX regressions, we developed a nomogram by using the R packages, “regplot”, “RMS” and “survival”. Calibration curves were used to assess the accuracy of the nomogram.

### Pathway enrichment analysis and gene set enrichment analysis

In this study, differentially expressed genes (DEGs) between high-risk and low-risk groups were identified using the R package “limma.” The threshold value was set to log2 |fold change|>1 and *p*-value < 0.05. We applied Gene Ontology (GO) and Kyoto Encyclopedia of Genes and Genomes (KEGG) analyses to elucidate molecular functions and key signaling pathways. At the same time, gene set enrichment analysis of EEA patients were performed to identify an association with disease phenotypes and pathways through R package clusterProfiler [21].

### Tumor microenvironment and immune cell infiltration profile

According to the proportion of immune cells and stromal cells in the tumor, an estimation algorithm was used to calculate the stromal score, immune score, estimated score and tumor purity. We applied TIMER, MCP-counter, CIBERSORT, QUANTISEQ, and xCell to calculate the proportion and abundance of tumor-infiltrating immune cells (TICs) in EEA [22]. Using the single-sample gene set enrichment analysis (ssGSEA) algorithm, we evaluated tumor-infiltrating immune cell infiltration and function [23]. An algorithm called Tumor Immune Dysfunction and Exclusion (TIDE) was used to predict the response to immunotherapy [24].

### Chemotherapeutic response and construction of the ceRNA network

The Cancer Cell Line Encyclopedia enables predictive modelling of anticancer drug sensitivity [25, 26]. The efficacy of chemotherapeutic drugs in EEA patients was predicted through the Genomics of Drug Sensitivity in Cancer (GDSC) database [27]. Drug sensitivity and gene expression profiling data of cancer cell lines in GDSC are integrated for investigation. The expression of each risk group in the gene set was performed by Spearman correlation analysis with the small molecule/drug sensitivity (IC50). The half maximal inhibitory concentration (IC50) was calculated by Prophetic software in R package.

According to the competitive endogenous RNA (ceRNA) theory, we constructed a network of lncRNAs, miRNAs, and mRNAs [28]. StarBase V2.0 was used to analyze the interaction between lncRNAs and miRNAs [29].

### Cell culture and quantitative real-time PCR analysis

HEC-1A is a human endometrial cancer cell line obtained from the Cell Bank of the Chinese Academy of Sciences. The HHUA cells were generously donated by the Department of Obstetrics and Gynecology, the First Affiliated Hospital of Shantou University Medical College. The cells were cultured in the presence of 10 μM Cu2+ and 10 nM Elesclomol for 48 hours, followed by microscopic observation and imaging. Cell proliferation was measured using the CCK8 kit (Beyotime, Shanghai, China). Subsequently, cellular RNA was extracted for further analysis. A control group was included, in which cells were cultured without the addition of Cu^2+^ and Elesclomol. TRIzol reagent was used to extract total RNA. Total RNA was reverse transcribed using PrimeScript reverse transcription reagent (Takara, Otsu, Shiga, Japan). In accordance with the manufacturer’s protocol, miRNA reverse transcription was performed using miRNA stem loop reverse transcription kit (Shanghai Sangong Biological Company, China). Quantitative PCR analysis was carried out using the 7500 Fast Real-Time PCR System instrument with TB Green Premix Ex Taq II (Takara, Japan) according to the manufacturer’s protocol. In each sample, the experiment was repeated three times and 2-Ct was used to calculate the RNA expression. Table 2 shows the primer sequences used in this study.

**Table 2:** Primer’s design.

**Primer**	**Sequence (5′ to 3′)**
ACOXL-AS1 F	TTCGGAGCTCTGGTTTCTGT
ACOXL-AS1 R	GGACTTATACCGACGCTCCA
β-actin F	GGCCAGGTCATCACCATTG
β-actin R	GGATGTCCACGTCACACTTCA
MTF1 F	ACTGGTGCCTTCCTCATCTGG
MTF1 R	CACTGTCCGTCGTCATCTTCATC
mir-421 F	CGCGGCCATCAACAGACATTAAT
mir-421 R	ATCCAGTGCAGGGTCCGAGG
mir-421 RT Primer	GTCGTATCCAGTGCAGGGTCCGAGGTATTCGCACTGGATACGACGCGCCC

### Lentiviral transfections and Western blotting

To establish human endometrial cancer cell lines with overexpression of ACOXL-AS1, we used ACOXL-AS1-expressing lentiviral vector (OE-ACOXL-AS1) and negative control lentiviral vector provided by Akey Biotechnology Co. LTD (Guangzhou, China). Human endometrial cancer cells were seeded in each well of a 6-well plate at a density of 1 × 105 cells per well. When the cell confluence reached 60% in each well, we added lentivirus (MOI = 10) to the cell culture medium. Then, we selected stable ACOXL-AS1-overexpressing cells with puromycin (3 μg/ml). Finally, we measured the expression level of ACOXL-AS1 in stable cells by qRT-PCR.

Cell lysis was performed with RIPA cell lysate (absin) and the lysates were centrifuged at 14000 g/min for 5 min. The supernatants were collected, and the protein concentration was determined by a BCA protein assay kit (Thermo Scientific). Equal amounts of protein were separated by SDS-PAGE and transferred to PVDF membranes. The membranes were incubated with the following primary antibodies: rabbit anti-MTF1 (1:1000; ab236401; Abcam), mouse anti-β-actin (1:1000; TA-09; ZSGB-BIO). Fluorescent antibody Anti-mouse/rabbit 800 was used as the secondary antibody. The signals were detected by Bio-Rad ChemiDoc™ MP Chemiluminescence/fluorescence imaging system (Bio-Rad) and quantified by ImageJ software (1.4.3.67, NIH).

### Statistical analysis

R (version 4.2.1) software was used for statistical analysis and visualization of the data. The following packages were used: “Maftools,” “ggplot2,” “limma,” “survminer,” “dplyr,” “plyr,” “survivalROC,” “clusterProfiler,” “ggplotify,” “cowplot,” “Hmisc,” “gridExtra,” “GSVA,” “corrplot,” “VennDiagram,” “pheatmap” and “tidyverse,”. When comparing two groups, the Wilcoxon test was used, and when comparing three groups, the ANOVA test was used. Prognosis was assessed using the Kaplan–Meier method and the Pearson method was used for correlation. We considered a *p*-value of 0.05 as statistically significant (^*^p < 0.05; ^**^p < 0.01; ^***^p < 0.001).

### Data and code availability statements

According to the TCGA project (https://portal.gdc.cancer.gov/) policies, all public access to these databases will be open. All code used in this manuscript are available at https://github.com/w28924461701/Cuproptosis-in-UCEC/.

## RESULTS

### Identification of CRLs and consensus clustering analysis

**Figure 1:**
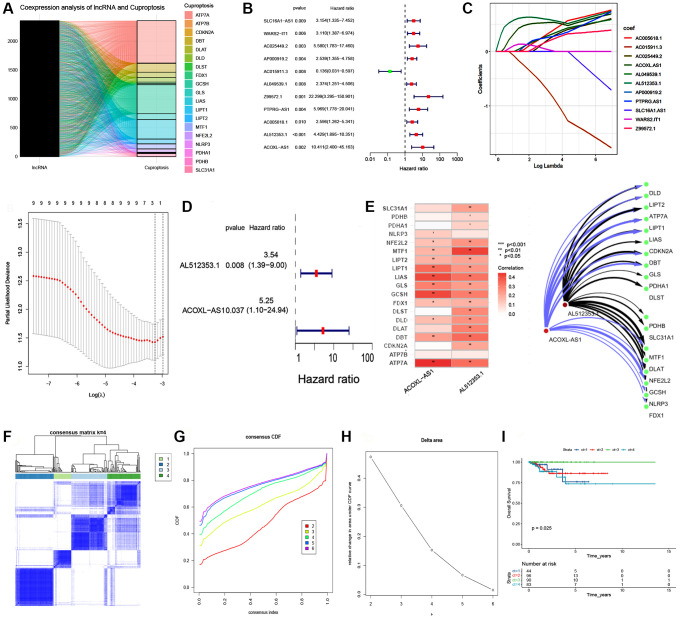
**Consensus clustering analysis of cuproptosis-related lncRNAs and LASSO regression model.** (**A**) Coexpression analysis of IncRNA and cuproptosis. (**B**) Univariate Cox regression analysis of cuproptosis-related lncRNAs. (**C**) LASSO regression model for the prognostic analysis of the lncRNA signature. (**D**) Forest plot visualing the results of multivariate Cox regression. (**E**) Correlation between two lncRNAs and 19 cuproptosis-related gene. Network visualization and analysis of lncRNAs and cuproptosis-related gene with Cytoscape. (**F**) Consensus matrix heatmap defining three clusters (k = 4) and their correlation area. (**G**, **H**) Cumulative distribution function (CDF) when k = 2–6. (**I**) Kaplan–Meier curve of the 4 clusters of patients with EEA in the TCGA cohort.

In the EEA cohort within TCGA, we obtained nineteen cuproptosis-related genes and 16,876 lncRNAs that were annotated by NCBI GenBank, Ensembl, and GENCODE. By co-expression analysis, 941 cuproptosis-related lncRNAs were found, and associations are visualized in the Sankey plot (Figure 1A). Eleven CRLs associated with prognosis were mined by univariate Cox regression analysis (Figure 1B, *P* < 0.05). The least absolute shrinkage and selection operator (LASSO) is an efficient gene selection method [30]. A total of 11 lncRNAs were selected to fit a LASSO regression model, the next step was to find the most appropriate values for λ (=0.00659) using 10-fold cross-validation (Figure 1C). Finally, two cuproptosis-related lncRNAs (AL512353.1, ACOXL-AS1) were identified by LASSO and Multivariate Cox regression analysis (Figure 1C, 1D). Cytoscape software was employed to visualize associations between lncRNAs and cuproptosis-related gene (Figure 1E).

Cluster analyses were conducted using the eleven prognosis-related lncRNAs. When EEA patients were divided into four subgroups, the clusters could be seen, and the subgroups showed good internal consistency and stability (Figure 1F–1H). Survival analysis revealed the prognostic significance of the clustering (Figure 1I, *P* = 0.025).

### Construction and validation of cuproptosis-related risk score and consensus clustering analysis

To further understand the prognostic role of the two cuproptosis-related lncRNAs, we randomly divided the dataset into training and validation groups. The training dataset was used to train a risk model, while the testing dataset was used to evaluate the performance of the model. A multivariate Cox regression analysis was then applied to the remaining genes to establish a prognostic signature in the training dataset. Regression coefficient in multivariate Cox regression analysis were derived from the training data, and were the parameters further refined using the combined dataset after passing the testing dataset. The risk score (RS) equation was defined as RS = (AL512353.1 × 1.264) + (ACOXL-AS1 × 1.658). According to the mathematical formula for calculating the risk score, patients were classified into a high risk or low risk group based on the median value of RS.

**Figure 2:**
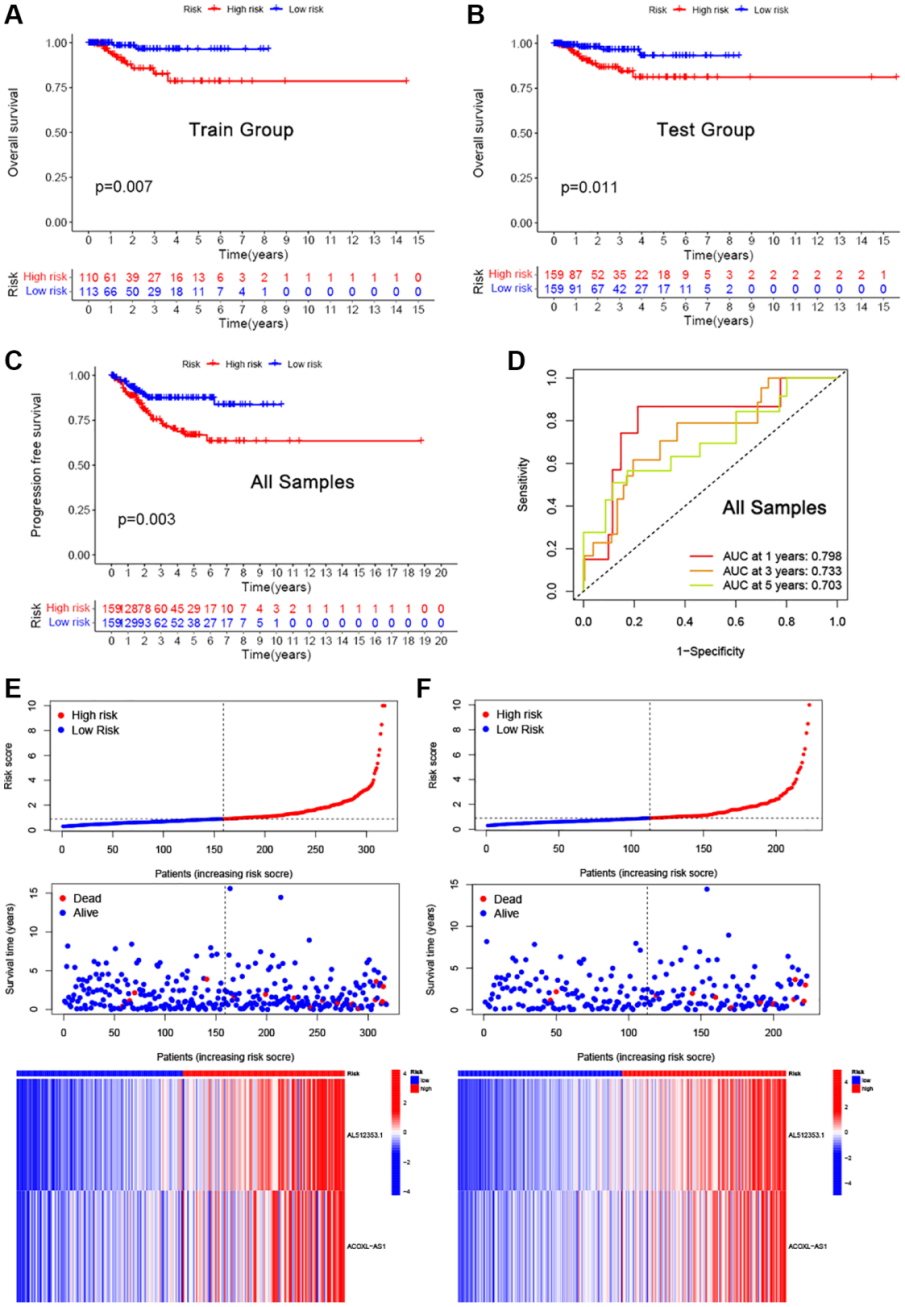
**Evaluation of the established cuproptosis-related lncRNAs signature in the training and test sets.** (**A**–**C**) Kaplan-Meier survival analysis in the training (**A**), validation (**B**) and all samples (**C**) sets. (**D**) ROC curves for risk scores at 1, 2, and 3 years. (**E**, **F**) Expression heat map, risk score distribution, and relapse status.

Kaplan-Meier survival analysis in the training, validation and all samples set showed that the overall survival (OS) of patients in the high-risk group predicted poor survival (Figure 2A–2C, *P* < 0.05). The model yielded better accuracy and calibrated survival estimates in predicting 1-, 3-, and 5-year survival (AUC: 0.703 to 0.798, Figure 2D). The layout of risk scores and survival status of EEA patients is shown in Figure 2E, 2F. In addition, the expression levels of the two cuproptosis-related lncRNAs between high- and low-risk groups are also displayed.

### Relationships between cuproptosis-related risk and clinicopathological features

**Figure 3:**
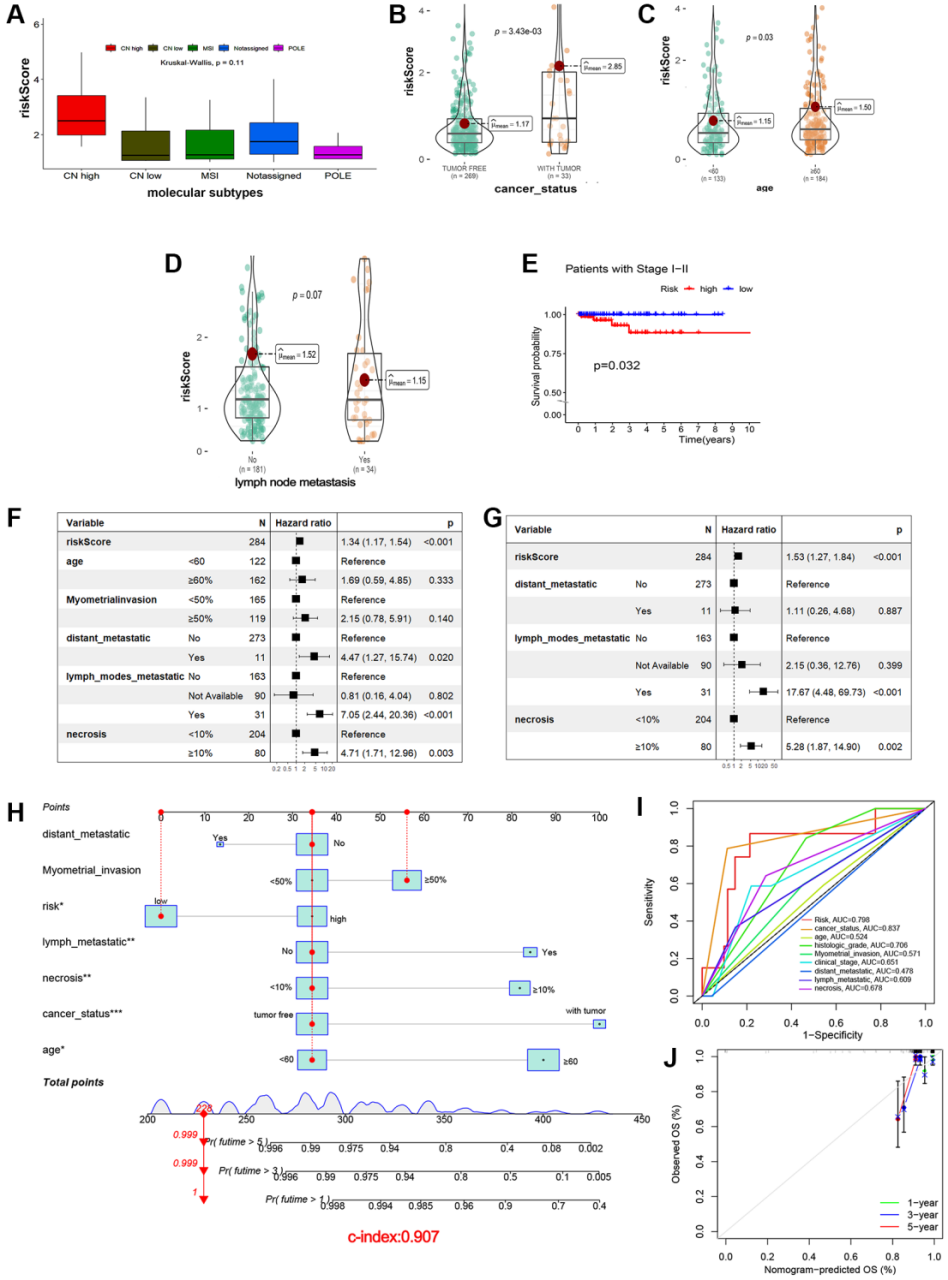
**Clinicopathological analysis and prognostic nomogram based on cuproptosis-related lncRNAs signature.** (**A**–**D**) Correlation between risk score and molecular subtypes (**A**), cancer status (**B**), age (**C**), and lymph node metastasis (**D**). (**E**) Correlation between risk score and survival probability in patients at stage I–II. (**F**, **G**) Univariate and multivariate Cox regression analysis. (**H**) Nomogram for predicting probabilities of EEA patient 1-, 3-, 5-year OS in the TCGA cohort. (**I**) ROC curves showing the predictive efficiency of the nomogram. (**J**) Calibration plots of 1-, 3-, and 5-year OS predicted by the nomograms.

The relationship between risk and clinicopathologic characteristics in EEA patients was further investigated. When comparing high-risk and low-risk groups with current clinical variables, we can use the chi-squared test to measure the association between categorical variables and the Wilcoxon rank-sum test to compare continuous variables. Risk classification was correlated with molecular subtypes, histologic grade, and myometrial invasion (*p* < 0.05), but other features did not obvious relevance (Figure 3A–3E, Table 3). The risk score was associated with molecular subtypes (*p* < 0.01), cancer status (*p* < 0.01) and age (*p* = 0.03), in EEA patients, POLE hypermutation and MSI subtypes have low risk values, while high CNV is associated with high-risk values (Figure 3A). For lymph node metastasis, there were no obvious differences in risk score between patients with lymph node metastasis and that without lymph node metastasis (Figure 3B–3D, *p* = 0.07). At the same time, in EAA patients in early-stage cancer (stage I, II), we also found that the patients in the predicted high-risk groups had shorter survival time than those in the low-risk groups (Figure 3E, *p* = 0.032).

**Table 3:** Association with clinical and risk group in endometrioid endometrial adenocarcinoma patients come from TCGA.

**Variable**	***N***	**High risk, *N* = 159**	**Low risk, *N* = 159**	***p*-value**
Molecular subtypes	247			0.001
CN high	14	6 (5%)	8 (6%)	
CN low	70	30 (25%)	40 (30%)	
MSI	50	15 (12%)	35 (27%)	
Notassigned	99	62 (53%)	37 (28%)	
POLE	14	4 (3%)	10 (7%)	
Age	317			0.079
<60		59 (37%)	74 (47%)	
≥60		100 (63%)	84 (53%)	
Histologic grade	318			0.031
Moderate		43 (27%)	49 (31%)	
Poor		85 (53%)	63 (40%)	
Well		31 (19%)	47 (30%)	
Myometrial invasion	285			0.038
<50%		89 (64%)	77 (52%)	
≥50%		49 (36%)	70 (48%)	
Clinical stage	318			0.68
Stage I–II		123 (77.4%)	127 (79.5%)	
Stage III–IV		36 (22.1%)	32 (19.8%)	
Distant metastatic	318			0.8
No		154 (97%)	153 (96%)	
Yes		5 (3.1%)	6 (3.8%)	
Lymph modes metastatic	318			0.86
No		89 (56%)	92 (58%)	
Yes		16 (10%)	18 (11%)	
Necrosis	318			0.5
<10%		111 (70%)	116 (73%)	
≥10%		48 (30%)	43 (27%)	

Pearson’s Chi-squared test; Wilcoxon rank sum test.

### Development of a nomogram following univariate and multivariate Cox analysis

To investigate the influence of clinicopathological features on prognosis of EEA patients, univariate and multivariate Cox proportional hazards analyses were employed, and results visualized as a forest plot. Univariate Cox analysis showed that risk Score, distant metastasis and necrosis were related to OS (Figure 3F* P* < 0.05). Then, multivariate Cox regression analyses demonstrated that risk Score and necrosis were also independently associated with OS (Figure 3G* P* < 0.05).

A nomogram model including all seven clinicopathologic factors was constructed to predict survival probability by adding all the points associated with the seven factors (Figure 3H). The higher the score, the greater the likelihood of death. The result showed that risk, age, lymph node metastasis, necrosis and cancer status were correlated with OS (*p* < 0.05). To validate reliability of the nomogram, the AUC values were calculated. Internal validation showed an AUC value of 0.816 (Figure 3I), a total C-index value for predicting OS of 0.907, and a C-index value of risk scores of 0.782. Calibration curves of the 1-, 3- and 5-year overall survival showed that the predicted OS was in good agreement with the observed OS (Figure 3J).

### Gene Ontology, KEGG pathway and gene Set enrichment analysis

**Figure 4:**
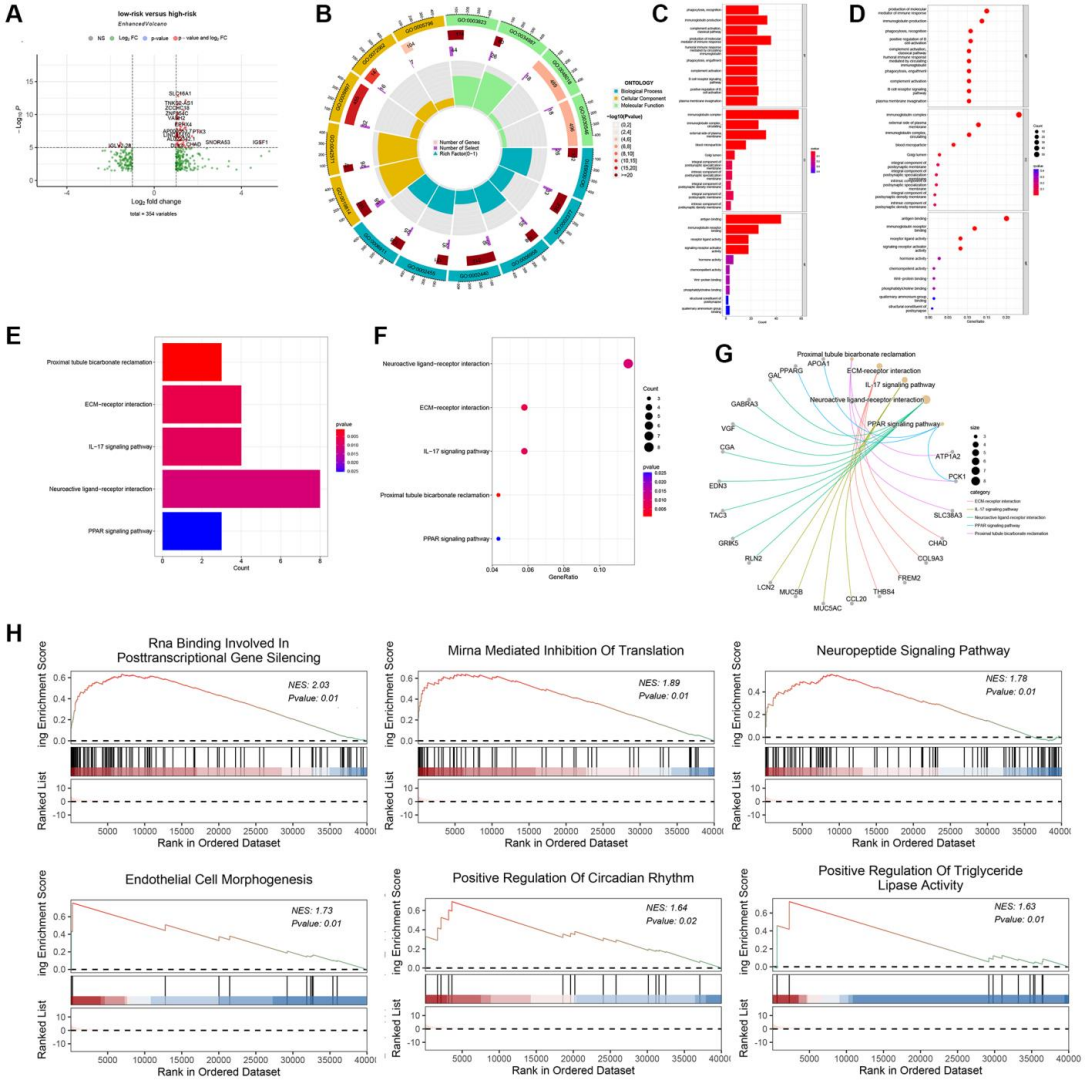
**Biological function, and mechanism analysis.** (**A**) Volcano plot of differentially-expressed genes (DEGs) between the high-risk group and low-risk group. (**B**) GO Circle plot (red, upregulated genes; blue, downregulated genes) show that Top 15 enriched GO terms in the BP category. (**C**) and (**D**) Plot of the enriched GO terms Go enrichment analysis for associated mRNAs with risk grouping. Y-axis represents the enriched GO terms; X-axis (**C**) represents the amount of the related mRNAs enriched in GO terms; X-axis (**D**) represents the ratio of the related mRNAs enriched in GO terms. (**E**) represents the amount of the related mRNAs enriched in KEGG pathways, X-axis. (**F**) represents the ratio of the related mRNAs enriched in KEGG pathways. (**G**) DEGs associated with the significant KEGG pathway. (**H**) GSEA showing the top six most significantly enriched signaling pathways.

Identification of differentially expressed genes (DEGs) can be used to gain mechanistic insights from diseases [31]. When R package limma was used to analyze differentially-expressed genes between the high-risk and low-risk groups, 354 genes (228 up-regulated and 126 down-regulated genes) showed significant changes (Figure 4A). The results of Gene Ontology (GO) analysis indicated that the differentially expressed genes (DEGs) were significantly enriched in various biological processes (BP), molecular functions (MF) and cellular components (CC). Specifically, the enriched BP terms included the production of molecular mediators of the immune response, while the enriched MF terms involved immunoglobulin complex formation, antigen binding, immunoglobulin receptor binding, and receptor-ligand activity (Figure 4B–4D). Based on the Kyoto Encyclopedia of Genes and Genomes (KEGG) pathway analysis, the differentially expressed genes (DEGs) were associated with multiple pathways, such as neuroactive ligand-receptor interactions, ECM receptor interactions, and IL-17 signaling pathways (Figure 4E–4G). Furthermore, GSEA identified that biological pathways including positive regulation of triglyceride lipase activity and neuropeptide signaling pathway were differentially enriched in the high and low risk groups (Figure 4H).

### Immune infiltration analysis and Immunotherapy

**Figure 5:**
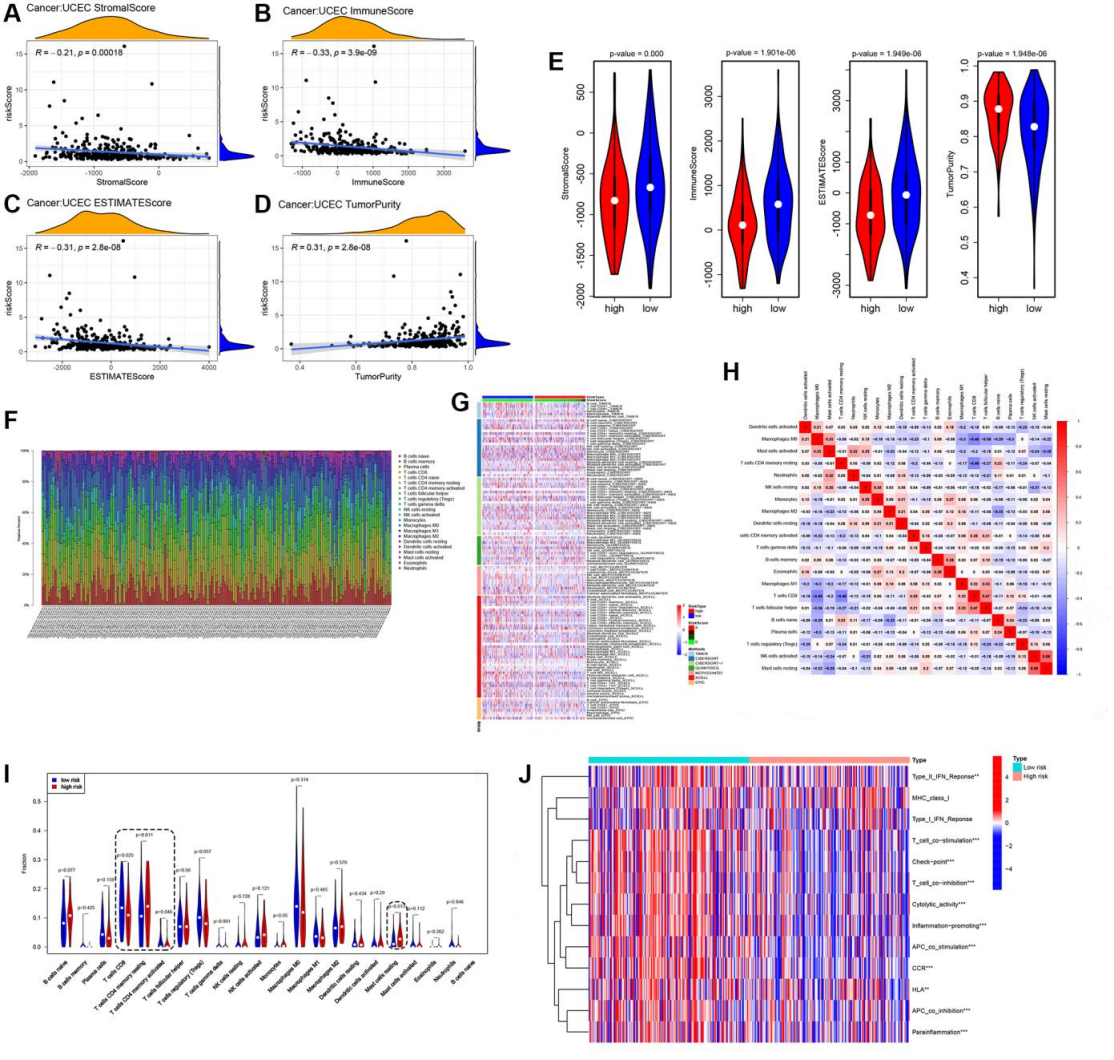
**Immune profile of cuproptosis lncRNAs based on EEA groups.** (**A**–**E**), Level of immune cell infiltration in the tumor, determined by ESTIMATE. (**F**, **G**) Infiltration situation of immune cells in each tumor sample. (**H**) Calculate of the association of 22 immune cell proportions. (**I**) CIBERSORT algorithm identifying the proportion of immune cells. (**J**) Scores of 13 immune-related functions by ssGSEA, ^*^p < 0.05, ^**^p < 0.01, ^***^p < 0.001.

The ESTIMATE algorithm was applied to infer tumor purity, immune score, and stromal score, which represent the level of immune cell infiltration in the tumor [32]. There was a correlation between the risk scores and both immune score, and stromal score (Figure 5A–5E, *p* < 0.01). The heat map showed infiltration of immune cells in each tumor sample, as determined by TIMER, CIBERSORT, quanTIseq, MCPcounter, XCell and Epic software (Figure 5F, 5G). The CIBERSORT algorithm was used to calculate the association of 22 immune cell proportions (Figure 5H). The CIBERSORT algorithm was also employed to identify the proportion of immune cells between high-risk and low-risk groups and showed that the signature in high-risk patients was associated with decreased CD8+ T lymphocyte infiltration, mast cells resting (Figure 5I). Meanwhile, ssGSEA was used to evaluate all 13 immune-related functions in the high- and low-risk groups, and the result showed obviously differences in immune check point, T cell co-stimulation, cytolytic activity, promoting of inflammation, and APC co stimulation, indicating a strong relationship between cuproptosis-related lncRNAs and immunity (Figure 5J).

Immune checkpoint inhibition is an immunotherapy method that blocks the binding of immune checkpoint proteins to chaperone proteins [33]. Gene expression analysis of immune checkpoint between the risk groups showed that PDCD1 and TIGIT were associated with risk group and tended to be expressed at low levels in the high-risk group (Supplementary Figure 1A). The scatter patterns of the top 15 most frequently mutated genes can be seen by looking at somatic variants, and it was found that the high-risk group had a lower rate of genetic alterations (94% vs. 97.35%, Supplementary Figure 1B, 1C) than the low-risk group. There were lower TMB values in the high-risk group, but higher tumor stemness and TIDE scores in the high-risk group. (Supplementary Figure 1D–1I).

### Prediction of chemotherapeutic response

**Figure 6:**
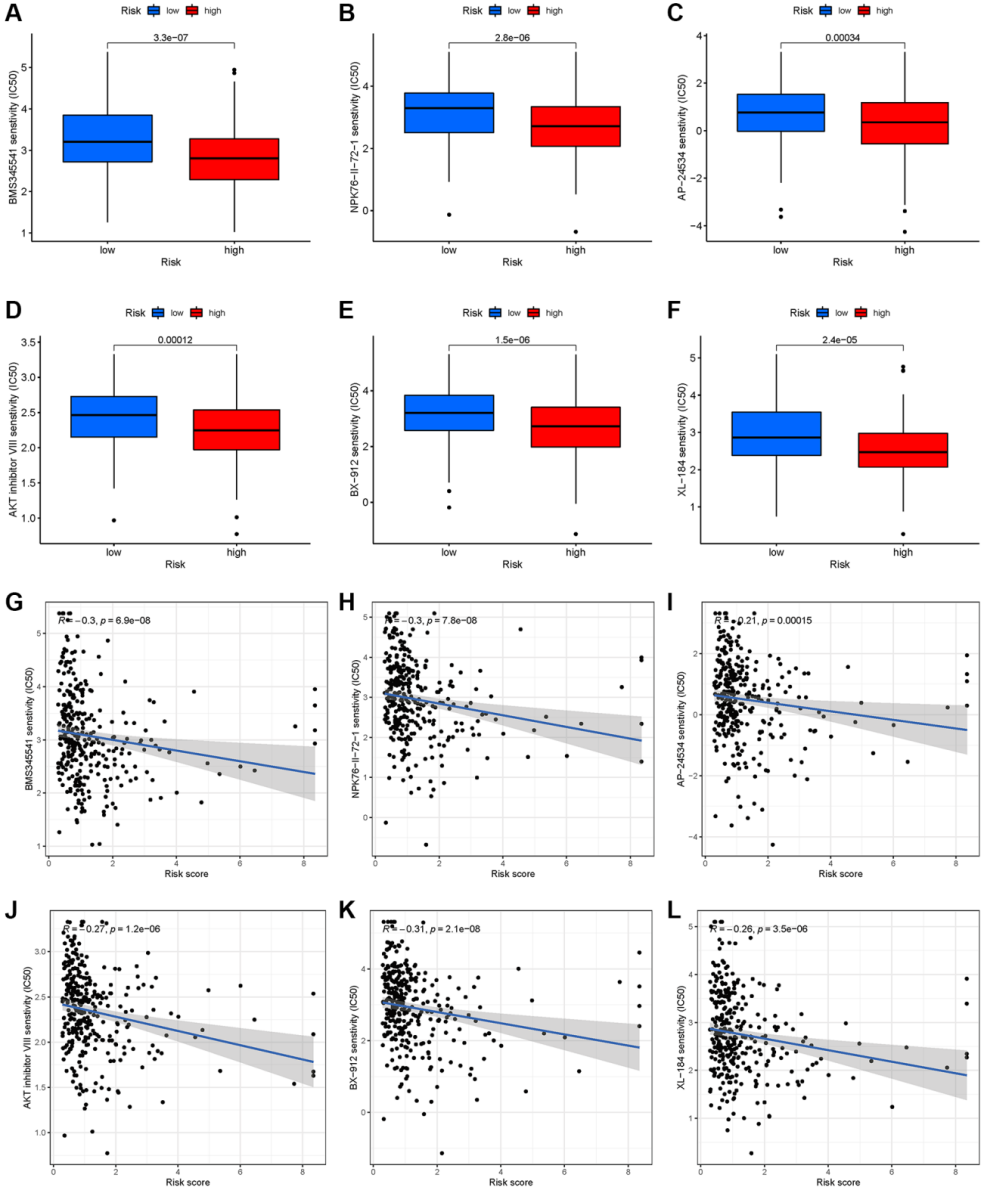
**Chemotherapy drug response prediction for EEA in high- and low-risk EEA patients.** (**A**) BMS345541. (**B**) NPK76-II-72-1. (**C**) AP-24534. (**D**) AKT inhibitor VIII. (**E**) BX-912. (**F**) XL-184. (**G**–**L**) Correlation analysis between risk group and 6 chemotherapy drugs.

By identifying molecular signatures of cancer, the GDSC database helps predict tumor’s response to antitumor therapy [34]. A large difference in IC50 was found between high-risk and low-risk groups for six chemotherapy agents, such as AKT inhibitors (Figure 6A–6F). Simultaneously, there was a correlation between six drugs and risk score (Figure 6G–6L, *p* < 0.001).

### Construction of LncRNA-miRNA-mRNA networks

**Figure 7:**
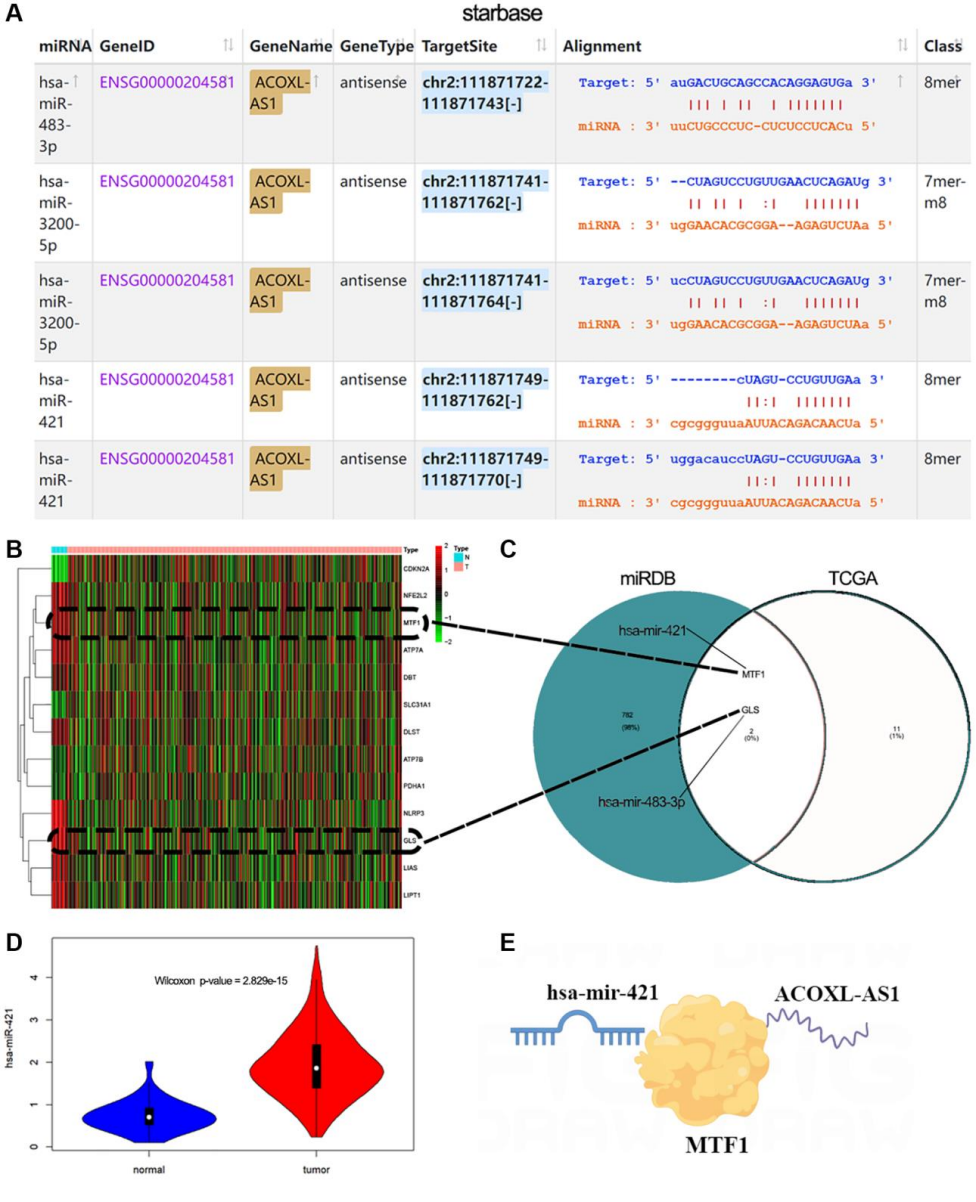
**Construction of LncRNA-miRNA-mRNA networks.** (**A**) StarBase V2.0 was used to analyze the interaction between lncRNAs and miRNAs. (**B**) Heatmap indicating cuproptosis-related genes show differential expression changes in normal and EEA tumor groups. (**C**) Venn diagram showing the common genes between TCGA and miRDB target genes. (**D**) miR-421 expression in normal or tumor group. (**E**) Schematic diagram for ACOXL-AS1-miR-421-MTF1 regulation.

Using starBase query results, miR-421, miR-483-3p, and miR-3200-5p can bind to their lncRNA ACOXL-AS1 targets (Figure 7A). Then, we conducted a differential comparison analysis of genes related to copper death, and the heatmap showed that 13 genes were differentially expressed between normal and tumor group in TCGA-EEA (Figure 7B). The online database miRDB provides functional annotation and predictions for miRNA targets [35]. Through the miRDB database, we found 3 miRNAs targeting 784 unique genes, and intersecting with 13 copper death-related genes in TCGA. Two of these genes, MTF1 and GLS were related to miR-421 and miR-483-3p, respectively (Figure 7C). At the same time, we also found that miR-421 was highly expressed in tumors (Figure 7D, *p* < 0.01). Thus, ACOXL-AS1 can serve as an endogenous “sponge” to regulate the expression of MTF1 by miR-421 (Figure 7E).

### Cuproptosis inhibited the cell proliferation and influenced on ACOXL-AS1, hsa-mir-421, and MTF1 expression in endometrial cancer cells

**Figure 8:**
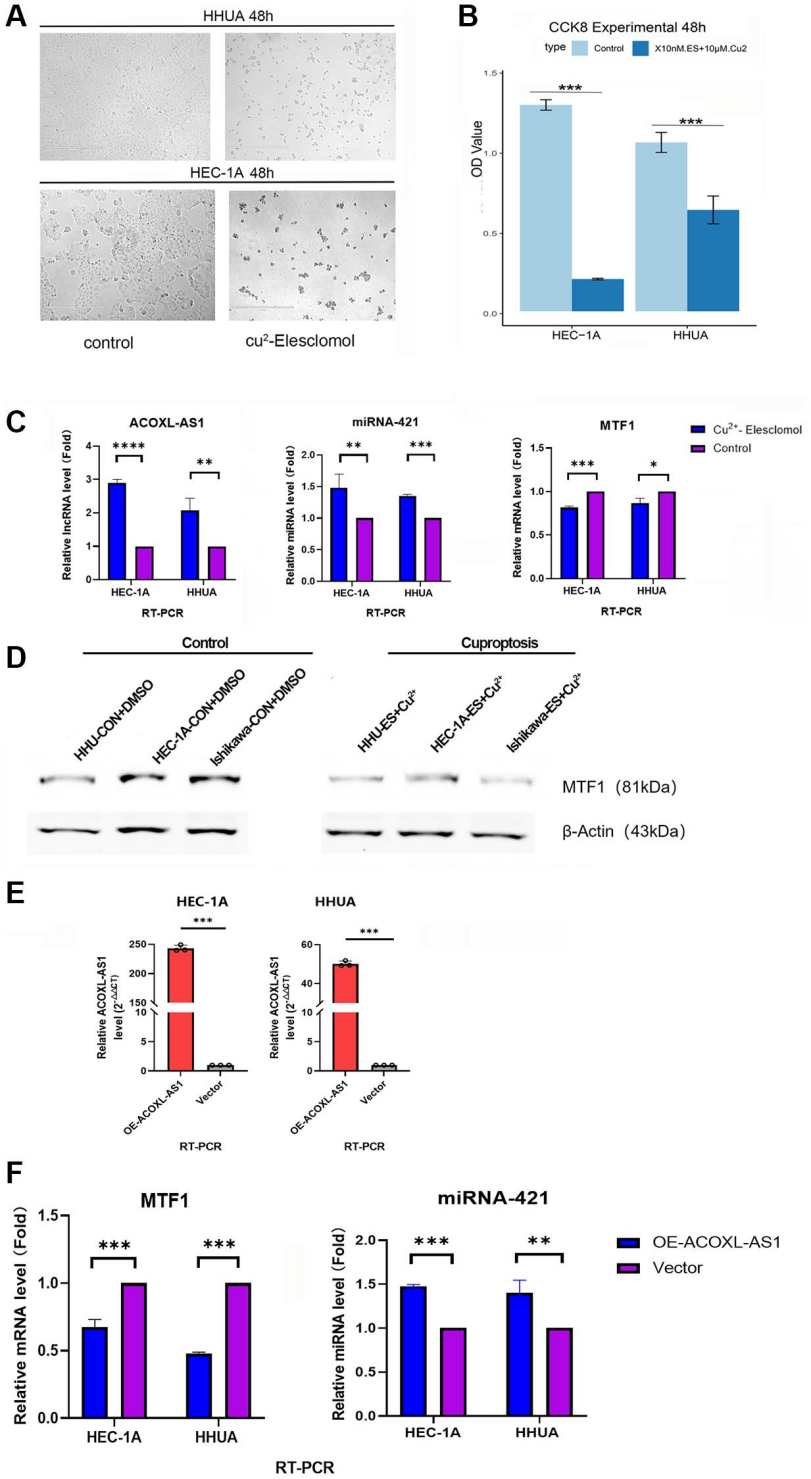
**Cuprotosis cell model and expression of ACOXL-AS1, mir-421 and MTF1 in endometrial cancer cells.** (**A**) Colony formation ability under differential conditions as determined by a colony formation assay. (**B**) Proliferation of endometrial cancer cells is detected by the Cell Counting Kit-8 assay. (**C**, **D**) Relative expression of ACOXL-AS1, mir-421 and MTF1 in endometrial cancer cells under different conditions. (**E**) overexpress the ACOXL-AS1 gene in HEC-1A and HHUA cells, while the “negative control lentiviral vector” is used as a reference (**F**) The impact of ACOXL-AS1 overexpression on the expression levels of MTF1 and miR-421, ^**^p < 0.01, ^***^p < 0.001, ^****^p < 0.0001.

To investigate the impact of cuproptosis on the growth of endometrial cancer cells, we established a copper-induced cell death model. When HHUA and HEC-1A, two endometrial cancer cell lines, were treated with Cu2+ plus Elesclomol, their growth was significantly inhibited in comparison to the control group (*p* < 0.001, Figure 8A, 8B).

In addition, the Cu2+ plus Elesclomol-treated group showed significantly higher gene expression levels for ACOXL-AS1 and hsa-mir-421 (*p* < 0.01, Figure 8C), while low expression levels were observed for MTF1 gene and protein in two endometrial cancer cell lines (*p* < 0.01, Figure 8C, 8D).

### Effect of ACOXL-AS1 overexpression on the expression of hsa-mir-421 and MTF1

To study the impact of ACOXL-AS1 on the expression of hsa-mir-421 and MTF1 in human endometrial cancer cell lines, two human endometrial cancer cell lines, HEC-1A and HHUA, were used in the study. Lentiviruses encoding ACOXL-AS1 cDNA (referred to as “ACOXL-AS1-OE”) and an empty vector were used to establish stable cells. The selection of stable cells was achieved using puromycin. ACOXL-AS1 expression in the stable cells was measured using quantitative Reverse Transcription Polymerase Chain Reaction (qRT-PCR). The qRT-PCR results showed that the expression of ACOXL-AS1 increased significantly in the ACOXL-AS1-overexpressed (“ACOXL-AS1-OE”) cells (Figure 8E). ACOXL-AS1-overexpressing cells showed increased expression of hsa-mir-421 and reduced MTF1 expression compared with controls (Figure 8F).

## DISCUSSION

Too much copper accumulation, will damage organs and lead to disease, such as hepatolenticular degeneration, but copper is essential [36]. Studies have shown elevated concentrations of copper in tumor tissue or serum in animal models and clinical patients with a variety of cancers [37]. Copper chelators are expected to be developed as adjuvant therapy for tumors in the future.

We systematically analyzed the influence of a cuproptosis related lncRNA signature on prognosis and immune features in EEA by LASSO, Cox regression modeling and consensus cluster analysis. A nomogram was constructed to evaluate predictive ability. More importantly, their immune properties and drug sensitivity were investigated based on this signature.

Clustering usually is the first step in data analysis [38]. To understand the value of cuproptosis-related lncRNAs in tumors, we first performed a consensus cluster analysis, and found that the cluster effect was appropriate, and showed the survival significance. To study whether the cuproptosis related lncRNA signature could predict prognosis in EEA cohorts, using the 11 cuproptosis-related lncRNAs, LASSO-Cox regression was conducted and two lncRNAs were identified to be associated with prognosis. A risk score model showed that cuproptosis related lncRNA signature was associated with survival in a training cohort and testing cohort, and the high-risk subtype was significantly associated with poor prognosis. These findings distinctly indicate that these signatures could provide prognostic biomarkers for patients with EEA.

The NCCN Guidelines for Uterine Neoplasms V.1.2021 indicates that G2-3 grade, age ≥60 years, deep muscle invasion and necrosis are high risk factors [39]. In addition, POLE mutations are associated with a high tumor mutational burden, which leads to an increased number of mutations in the DNA of the tumor cells. This high mutation rate can result in the production of abnormal proteins that can be recognized by the immune system, leading to a better prognosis and a lower risk of recurrence. Similarly, MSI is associated with defects in DNA repair mechanisms, leading to an increased number of mutations. This subtype is also associated with a better prognosis and a lower risk of recurrence. Our analysis of clinicopathological factors has shown that risk classification is correlated with molecular subtypes, histologic grade, and myometrial invasion. Univariate and multivariate analysis showed that risk score remains an independent prognostic factor, along with age, cancer status, lymph metastasis and necrosis. Therefore, our results suggest that risk scores of cuproptosis-related lncRNA signature can be poor prognostic factors. Nomograms are a common tool for estimating prognosis in oncology and medicine, and are designed to help physicians assess risk [40]. Calibration curves and C-indices are reliable indicators for evaluating nomogram models [41, 42]. That is to say, the larger the C-index, the more accurate the prognosis prediction. Our study shows that risk score, lymph node metastasis, necrosis and age are associated with poor survival. The total C-index and calibration curves for the 1-, 3- and 5-year overall survival demonstrated the good reliability of this model.

The immune response involves the recognition and elimination of cancer cells by various immune cells and molecules. Immunoglobulins, also known as antibodies, play a critical role in the immune response by recognizing and binding to specific antigens on the surface of cancer cells [43]. The formation of immunoglobulin complexes and the binding of immunoglobulin receptors to antigens are key steps in the immune response against tumors [44]. In tumors, aberrant expression or dysregulation of these interactions can contribute to tumor growth, invasion, and metastasis. Our present analysis utilizing Go methodology precisely identifies these molecular functions. These findings provide evidence that the risk stratification of long non-coding RNAs (lncRNAs) linked to copper-induced cell death is correlated with immune function.

Studies have shown that neuroactive ligand-receptor interactions play a crucial role in regulating various physiological processes, including cell proliferation, differentiation, and apoptosis [45, 46]. ECM (extracellular matrix) receptor interactions also play a vital role in tumor development and progression. ECM proteins, such as collagens, laminins, and fibronectin, provide structural support for cells and facilitate cell signaling [47].

Adipose triglyceride lipase activity regulates cancer cell proliferation and cancer invasion [48]. Overall, these three signaling pathways – neuroactive ligand-receptor interactions, ECM receptor interactions, and IL-17 signaling pathways – all play important roles in tumor development and progression by KEGG pathway and gene set enrichment analysis. These studies also suggest that cuproptosis related lncRNAs promote tumor progression.

A comprehensive understanding of the characteristics of the tumor immune microenvironment is essential for optimizing the efficacy of immunotherapy [49]. Tumor purity, immune score, and stromal score are three crucial factors that can exert significant influence on tumor progression [50]. Research has revealed that higher levels of tumor purity are generally associated with a poorer prognosis, as this suggests more aggressive cancer cells and a greater potential for metastasis. Conversely, a higher immune score has been positively correlated with better outcomes, as this indicates an effective recognition and attack of cancer cells by the immune system [51, 52]. Similarly, higher stromal scores have been associated with a worse prognosis, indicating a well-supported and thriving tumor with the capacity to grow and spread [52]. And just like the results above, we found that patients in the high-risk group have a lower interstitial score and immune score than those in the low-risk group.

CD8+ T lymphocytes, also known as cytotoxic T cells, are a type of immune cell that can recognize and kill cancer cells [53]. Therapies that target immune checkpoint markers, such as monoclonal antibodies that block PD-L1 and CTLA-4, have shown promise in treating a variety of cancers, particularly those that are associated with a high level of immune checkpoint marker expression. These therapies have been shown to increase the infiltration of CD8+ T cells into tumors, leading to improved patient outcomes [54]. Our results showed the signature in high-risk patients is associated with decreased CD8+ T lymphocyte infiltration, and immune checkpoint markers (PD-L1). The study suggests that it led to poorer tumor outcomes, such as increased tumor growth, metastasis, and decreased survival rates.

The use of tumor mutation burden and microsatellite instability to predict the response to immunotherapy has been included in the latest NCCN 2022 guidelines. In the high-risk group, TMB values were lower, but tumor stemness and TIDE scores were higher.

The above results indicate that high-risk EEA patients do not respond to or can escape from immunotherapy.

To find effective treatment method for high-risk EEA patients with a cuproptosis-related lncRNA signature, we used the GDSC database to predict a patient’s response to antitumor therapy. We found six types of compounds to be effective for patients in the high-risk group, particularly AKT inhibitors.

At the same time, we constructed a ceRNA network of cuproptosis-related lncRNAs-miRNAs-mRNAs by bioinformatics methods. An *in vitro* cell experiment was also conducted to determine the gene expression relationship of the ceRNA network. These results are helpful for further study of the molecular mechanisms of cuproptosis related lncRNA signature.

However, our study still has some limitations, and a large number of clinical samples will be needed to verify our results and confirm whether the conclusions to guide clinical treatment.

## CONCLUSION

We identify a cuproptosis-related lncRNA signature that predicts poor outcomes and is associated with a decrease of CD8+ T lymphocyte infiltration in patients with EEA. AKT inhibitors may provide therapeutic benefits.

## Supplementary Materials

Supplementary Figure 1

## References

[1] Guan J, Xie L, Luo X, Yang B, Zhang H, Zhu Q, Chen X. The prognostic significance of estrogen and progesterone receptors in grade I and II endometrioid endometrial adenocarcinoma: hormone receptors in risk stratification. J Gynecol Oncol. 2019; 30:e13. 10.3802/jgo.2019.30.e13 30479097 PMC6304404

[2] McVicker L, Cardwell CR, Edge L, McCluggage WG, Quinn D, Wylie J, McMenamin ÚC. Survival outcomes in endometrial cancer patients according to diabetes: a systematic review and meta-analysis. BMC Cancer. 2022; 22:427. 10.1186/s12885-022-09510-7 35439978 PMC9019948

[3] Murali R, Delair DF, Bean SM, Abu-Rustum NR, Soslow RA. Evolving Roles of Histologic Evaluation and Molecular/Genomic Profiling in the Management of Endometrial Cancer. J Natl Compr Canc Netw. 2018; 16:201–9. 10.6004/jnccn.2017.7066 29439179 PMC6639790

[4] Chen L, Min J, Wang F. Copper homeostasis and cuproptosis in health and disease. Signal Transduct Target Ther. 2022; 7:378. 10.1038/s41392-022-01229-y 36414625 PMC9681860

[5] Wang Y, Zhang L, Zhou F. Cuproptosis: a new form of programmed cell death. Cell Mol Immunol. 2022; 19:867–8. 10.1038/s41423-022-00866-1 35459854 PMC9338229

[6] Tsvetkov P, Coy S, Petrova B, Dreishpoon M, Verma A, Abdusamad M, Rossen J, Joesch-Cohen L, Humeidi R, Spangler RD, Eaton JK, Frenkel E, Kocak M, et al. Copper induces cell death by targeting lipoylated TCA cycle proteins. Science. 2022; 375:1254–61. 10.1126/science.abf0529 35298263 PMC9273333

[7] Tang D, Chen X, Kroemer G. Cuproptosis: a copper-triggered modality of mitochondrial cell death. Cell Res. 2022; 32:417–8. 10.1038/s41422-022-00653-7 35354936 PMC9061796

[8] Xie J, Yang Y, Gao Y, He J. Cuproptosis: mechanisms and links with cancers. Mol Cancer. 2023; 22:46. 10.1186/s12943-023-01732-y 36882769 PMC9990368

[9] Bridges MC, Daulagala AC, Kourtidis A. LNCcation: lncRNA localization and function. J Cell Biol. 2021; 220:e202009045. 10.1083/jcb.202009045 33464299 PMC7816648

[10] Tan YT, Lin JF, Li T, Li JJ, Xu RH, Ju HQ. LncRNA-mediated posttranslational modifications and reprogramming of energy metabolism in cancer. Cancer Commun (Lond). 2021; 41:109–20. 10.1002/cac2.12108 33119215 PMC7896749

[11] Hutter C, Zenklusen JC. The Cancer Genome Atlas: Creating Lasting Value beyond Its Data. Cell. 2018; 173:283–5. 10.1016/j.cell.2018.03.042 29625045

[12] Ritchie ME, Phipson B, Wu D, Hu Y, Law CW, Shi W, Smyth GK. limma powers differential expression analyses for RNA-sequencing and microarray studies. Nucleic Acids Res. 2015; 43:e47. 10.1093/nar/gkv007 25605792 PMC4402510

[13] Wu H, Zhang J. Decreased expression of TFAP2B in endometrial cancer predicts poor prognosis: A study based on TCGA data. Gynecol Oncol. 2018; 149:592–7. 10.1016/j.ygyno.2018.03.057 29602546

[14] Mayakonda A, Lin DC, Assenov Y, Plass C, Koeffler HP. Maftools: efficient and comprehensive analysis of somatic variants in cancer. Genome Res. 2018; 28:1747–56. 10.1101/gr.239244.118 30341162 PMC6211645

[15] Malta TM, Sokolov A, Gentles AJ, Burzykowski T, Poisson L, Weinstein JN, Kamińska B, Huelsken J, Omberg L, Gevaert O, Colaprico A, Czerwińska P, Mazurek S, et al, and Cancer Genome Atlas Research Network. Machine Learning Identifies Stemness Features Associated with Oncogenic Dedifferentiation. Cell. 2018; 173:338–54.e15. 10.1016/j.cell.2018.03.034 29625051 PMC5902191

[16] Zeng D, Ye Z, Shen R, Yu G, Wu J, Xiong Y, Zhou R, Qiu W, Huang N, Sun L, Li X, Bin J, Liao Y, et al. IOBR: Multi-Omics Immuno-Oncology Biological Research to Decode Tumor Microenvironment and Signatures. Front Immunol. 2021; 12:687975. 10.3389/fimmu.2021.687975 34276676 PMC8283787

[17] Li SR, Bu LL, Cai L. Cuproptosis: lipoylated TCA cycle proteins-mediated novel cell death pathway. Signal Transduct Target Ther. 2022; 7:158. 10.1038/s41392-022-01014-x 35562341 PMC9106713

[18] Wilkerson MD, Hayes DN. ConsensusClusterPlus: a class discovery tool with confidence assessments and item tracking. Bioinformatics. 2010; 26:1572–3. 10.1093/bioinformatics/btq170 20427518 PMC2881355

[19] Tibshirani R. The lasso method for variable selection in the Cox model. Stat Med. 1997; 16:385–95. 10.1002/(sici)1097-0258(19970228)16:4<385::aid-sim380>3.0.co;2-3 9044528

[20] Iasonos A, Schrag D, Raj GV, Panageas KS. How to build and interpret a nomogram for cancer prognosis. J Clin Oncol. 2008; 26:1364–70. 10.1200/JCO.2007.12.9791 18323559

[21] Wu T, Hu E, Xu S, Chen M, Guo P, Dai Z, Feng T, Zhou L, Tang W, Zhan L, Fu X, Liu S, Bo X, Yu G. clusterProfiler 4.0: A universal enrichment tool for interpreting omics data. Innovation (Camb). 2021; 2:100141. 10.1016/j.xinn.2021.100141 34557778 PMC8454663

[22] Li T, Fu J, Zeng Z, Cohen D, Li J, Chen Q, Li B, Liu XS. TIMER2.0 for analysis of tumor-infiltrating immune cells. Nucleic Acids Res. 2020; 48:W509–14. 10.1093/nar/gkaa407 32442275 PMC7319575

[23] Zhuang W, Sun H, Zhang S, Zhou Y, Weng W, Wu B, Ye T, Huang W, Lin Z, Shi L, Shi K. An immunogenomic signature for molecular classification in hepatocellular carcinoma. Mol Ther Nucleic Acids. 2021; 25:105–15. 10.1016/j.omtn.2021.06.024 34401208 PMC8332372

[24] Jiang P, Gu S, Pan D, Fu J, Sahu A, Hu X, Li Z, Traugh N, Bu X, Li B, Liu J, Freeman GJ, Brown MA, et al. Signatures of T cell dysfunction and exclusion predict cancer immunotherapy response. Nat Med. 2018; 24:1550–8. 10.1038/s41591-018-0136-1 30127393 PMC6487502

[25] Geeleher P, Cox NJ, Huang RS. Clinical drug response can be predicted using baseline gene expression levels and in vitro drug sensitivity in cell lines. Genome Biol. 2014; 15:R47. 10.1186/gb-2014-15-3-r47 24580837 PMC4054092

[26] Barretina J, Caponigro G, Stransky N, Venkatesan K, Margolin AA, Kim S, Wilson CJ, Lehár J, Kryukov GV, Sonkin D, Reddy A, Liu M, Murray L, et al. The Cancer Cell Line Encyclopedia enables predictive modelling of anticancer drug sensitivity. Nature. 2012; 483:603–7. 10.1038/nature11003 22460905 PMC3320027

[27] Piper M, Gronostajski R, Messina G. Nuclear Factor One X in Development and Disease. Trends Cell Biol. 2019; 29:20–30. 10.1016/j.tcb.2018.09.003 30287093

[28] Ala U. Competing Endogenous RNAs, Non-Coding RNAs and Diseases: An Intertwined Story. Cells. 2020; 9:1574. 10.3390/cells9071574 32605220 PMC7407898

[29] Li JH, Liu S, Zhou H, Qu LH, Yang JH. starBase v2.0: decoding miRNA-ceRNA, miRNA-ncRNA and protein-RNA interaction networks from large-scale CLIP-Seq data. Nucleic Acids Res. 2014; 42:D92–7. 10.1093/nar/gkt1248 24297251 PMC3964941

[30] Xiong Y, Ling QH, Han F, Liu QH. An efficient gene selection method for microarray data based on LASSO and BPSO. BMC Bioinformatics. 2019; 20:715. 10.1186/s12859-019-3228-0 31888444 PMC6936154

[31] Xiong H, Guo H, Xie Y, Zhao L, Gu J, Zhao S, Li J, Liu L. RNAseq analysis reveals pathways and candidate genes associated with salinity tolerance in a spaceflight-induced wheat mutant. Sci Rep. 2017; 7:2731. 10.1038/s41598-017-03024-0 28578401 PMC5457441

[32] Yoshihara K, Shahmoradgoli M, Martínez E, Vegesna R, Kim H, Torres-Garcia W, Treviño V, Shen H, Laird PW, Levine DA, Carter SL, Getz G, Stemke-Hale K, et al. Inferring tumour purity and stromal and immune cell admixture from expression data. Nat Commun. 2013; 4:2612. 10.1038/ncomms3612 24113773 PMC3826632

[33] Bagchi S, Yuan R, Engleman EG. Immune Checkpoint Inhibitors for the Treatment of Cancer: Clinical Impact and Mechanisms of Response and Resistance. Annu Rev Pathol. 2021; 16:223–49. 10.1146/annurev-pathol-042020-042741 33197221

[34] Yang W, Soares J, Greninger P, Edelman EJ, Lightfoot H, Forbes S, Bindal N, Beare D, Smith JA, Thompson IR, Ramaswamy S, Futreal PA, Haber DA, et al. Genomics of Drug Sensitivity in Cancer (GDSC): a resource for therapeutic biomarker discovery in cancer cells. Nucleic Acids Res. 2013; 41:D955–61. 10.1093/nar/gks1111 23180760 PMC3531057

[35] Wang X. miRDB: a microRNA target prediction and functional annotation database with a wiki interface. RNA. 2008; 14:1012–7. 10.1261/rna.965408 18426918 PMC2390791

[36] Bandmann O, Weiss KH, Kaler SG. Wilson's disease and other neurological copper disorders. Lancet Neurol. 2015; 14:103–13. 10.1016/S1474-4422(14)70190-5 25496901 PMC4336199

[37] Wang F, Jiao P, Qi M, Frezza M, Dou QP, Yan B. Turning tumor-promoting copper into an anti-cancer weapon via high-throughput chemistry. Curr Med Chem. 2010; 17:2685–98. 10.2174/092986710791859315 20586723 PMC3786439

[38] Guijo-Rubio D, Duran-Rosal AM, Gutierrez PA, Troncoso A, Hervas-Martinez C. Time-Series Clustering Based on the Characterization of Segment Typologies. IEEE Trans Cybern. 2021; 51:5409–22. 10.1109/TCYB.2019.2962584 31945011

[39] Abu-Rustum NR, Yashar CM, Bradley K, Campos SM, Chino J, Chon HS, Chu C, Cohn D, Crispens MA, Damast S, Diver E, Fisher CM, Frederick P, et al. NCCN Guidelines® Insights: Uterine Neoplasms, Version 3.2021. J Natl Compr Canc Netw. 2021; 19:888–95. 10.6004/jnccn.2021.0038 34416706

[40] Balachandran VP, Gonen M, Smith JJ, DeMatteo RP. Nomograms in oncology: more than meets the eye. Lancet Oncol. 2015; 16:e173–80. 10.1016/S1470-2045(14)71116-7 25846097 PMC4465353

[41] Vickers AJ, Cronin AM. Everything you always wanted to know about evaluating prediction models (but were too afraid to ask). Urology. 2010; 76:1298–301. 10.1016/j.urology.2010.06.019 21030068 PMC2997853

[42] Huitzil-Melendez FD, Capanu M, O'Reilly EM, Duffy A, Gansukh B, Saltz LL, Abou-Alfa GK. Advanced hepatocellular carcinoma: which staging systems best predict prognosis? J Clin Oncol. 2010; 28:2889–95. 10.1200/JCO.2009.25.9895 20458042 PMC3651603

[43] Newton K, Dixit VM. Signaling in innate immunity and inflammation. Cold Spring Harb Perspect Biol. 2012; 4:a006049. 10.1101/cshperspect.a006049 22296764 PMC3282411

[44] Fridman WH, Meylan M, Petitprez F, Sun CM, Italiano A, Sautès-Fridman C. B cells and tertiary lymphoid structures as determinants of tumour immune contexture and clinical outcome. Nat Rev Clin Oncol. 2022; 19:441–57. 10.1038/s41571-022-00619-z 35365796

[45] Zheng R, Iwase A, Shen R, Goodman OB Jr, Sugimoto N, Takuwa Y, Lerner DJ, Nanus DM. Neuropeptide-stimulated cell migration in prostate cancer cells is mediated by RhoA kinase signaling and inhibited by neutral endopeptidase. Oncogene. 2006; 25:5942–52. 10.1038/sj.onc.1209586 16652149

[46] Kasprzak A, Adamek A. The Neuropeptide System and Colorectal Cancer Liver Metastases: Mechanisms and Management. Int J Mol Sci. 2020; 21:3494. 10.3390/ijms21103494 32429087 PMC7279011

[47] Sainio A, Järveläinen H. Extracellular matrix-cell interactions: Focus on therapeutic applications. Cell Signal. 2020; 66:109487. 10.1016/j.cellsig.2019.109487 31778739

[48] Wang YY, Attané C, Milhas D, Dirat B, Dauvillier S, Guerard A, Gilhodes J, Lazar I, Alet N, Laurent V, Le Gonidec S, Biard D, Hervé C, et al. Mammary adipocytes stimulate breast cancer invasion through metabolic remodeling of tumor cells. JCI Insight. 2017; 2:e87489. 10.1172/jci.insight.87489 28239646 PMC5313068

[49] Chew V, Toh HC, Abastado JP. Immune microenvironment in tumor progression: characteristics and challenges for therapy. J Oncol. 2012; 2012:608406. 10.1155/2012/608406 22927846 PMC3423944

[50] Chen F, Zhuang X, Lin L, Yu P, Wang Y, Shi Y, Hu G, Sun Y. New horizons in tumor microenvironment biology: challenges and opportunities. BMC Med. 2015; 13:45. 10.1186/s12916-015-0278-7 25857315 PMC4350882

[51] Xu Q, Chen S, Hu Y, Huang W. Landscape of Immune Microenvironment Under Immune Cell Infiltration Pattern in Breast Cancer. Front Immunol. 2021; 12:711433. 10.3389/fimmu.2021.711433 34512634 PMC8429934

[52] Calon A, Lonardo E, Berenguer-Llergo A, Espinet E, Hernando-Momblona X, Iglesias M, Sevillano M, Palomo-Ponce S, Tauriello DV, Byrom D, Cortina C, Morral C, Barceló C, et al. Stromal gene expression defines poor-prognosis subtypes in colorectal cancer. Nat Genet. 2015; 47:320–9. 10.1038/ng.3225 25706628

[53] Galon J, Costes A, Sanchez-Cabo F, Kirilovsky A, Mlecnik B, Lagorce-Pagès C, Tosolini M, Camus M, Berger A, Wind P, Zinzindohoué F, Bruneval P, Cugnenc PH, et al. Type, density, and location of immune cells within human colorectal tumors predict clinical outcome. Science. 2006; 313:1960–4. 10.1126/science.1129139 17008531

[54] Pardoll DM. The blockade of immune checkpoints in cancer immunotherapy. Nat Rev Cancer. 2012; 12:252–64. 10.1038/nrc3239 22437870 PMC4856023

