# Supplementary material for: Two lncRNA signatures with cuproptosis as a novel prognostic model and clinicopathological value for endometrioid endometrial adenocarcinoma
Source: Aging (Albany NY). 2023 Dec 11;15(23):14242–62. doi: 10.18632/aging.205299 (PMC10756113; doi:10.18632/aging.205299)
Supplement: Supplementary Figure 1 [file aging-15-205299-s001.pdf]

SUPPLEMENTARY FIGURE

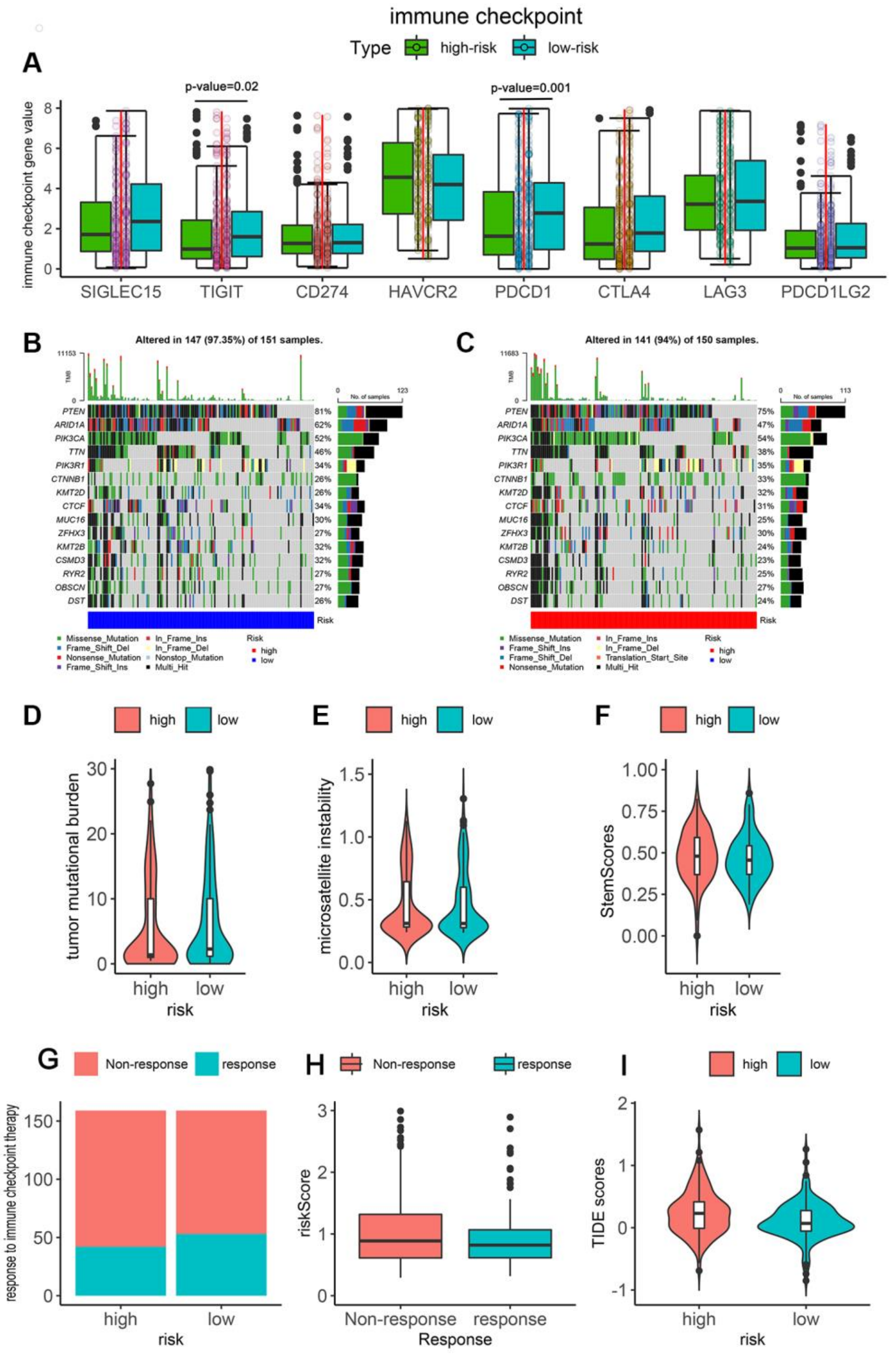

Supplementary Figure 1. Response to immune checkpoint therapy, tumor stemness score. (**A**) Immune checkpoint. (**B**, **C**) Oncoplots for the top 15 mutated genes in low- (**B**) and high-risk (**C**) groups. (**D**–**H**) Correlation between risk score and tumor mutation burden (TMB) (**D**), microsatellite instability (MSI) (**E**), stemness score (**F**), response to Immune checkpoint therapy (**G**, **H**). (**I**) Correlation between risk score and TIDE score.
